# Prevalence and Clinical Correlates of Internet Addiction Symptoms and Their Association With Quality of Life in Adolescents With Major Depressive Disorder: A Multicenter Cross-Sectional Study

**DOI:** 10.3389/fpsyt.2022.819704

**Published:** 2022-04-25

**Authors:** Song Wang, Lei Xia, Jiawei Wang, Xiaoping Yuan, Yudong Shi, Xixin Wang, Xiaoyue Li, Yu Hu, Yulong Zhang, Yating Yang, Feng Geng, Zhiwei Liu, Changhao Chen, Xiangwang Wen, Xiangfen Luo, Fei Gao, Huanzhong Liu

**Affiliations:** ^1^School of Mental Health and Psychological Sciences, Anhui Medical University, Hefei, China; ^2^Department of Psychiatry, Chaohu Hospital of Anhui Medical University, Hefei, China; ^3^Anhui Psychiatric Center, Hefei, China; ^4^Department of Psychiatry, Bozhou People’s Hospital, Bozhou, China; ^5^Department of Psychiatry, Hefei Fourth People’s Hospital, Hefei, China; ^6^Department of Psychiatry, Fuyang Third People’s Hospital, Fuyang, China; ^7^Department of Psychiatry, Suzhou Second People’s Hospital, Suzhou, China; ^8^Department of Psychiatry, Ma’anshan Fourth People’s Hospital, Ma’anshan, China; ^9^Department of Psychiatry, The Second Affiliated Hospital of Bengbu Medical College, Bengbu, China; ^10^Centre for Cognitive and Brain Sciences, University of Macau, Macao, Macao SAR, China

**Keywords:** major depressive disorder, internet addiction, adolescents, quality of life, China

## Abstract

**Background:**

Internet addiction (IA) symptoms are common among adolescents and negatively impact their academic performance and development. These symptoms are also associated with lower quality of life (QOL) and increased suicidality. This study aimed to examine the prevalence and the sociodemographic and clinical correlates of IA symptoms in adolescents with major depressive disorder (MDD) and their association with QOL.

**Methods:**

This cross-sectional study was conducted in three general and four psychiatric hospitals in Anhui Province, China, from January to July 2021. Sociodemographic and clinical data were collected. The Internet Addiction Test (IAT), Center for Epidemiologic Studies of Depression Symptom Scale (CES-D), and World Health Organization Quality of Life Brief version (WHOQOL-BREF) were utilized to examine IA, depressive symptoms, and QOL, respectively.

**Results:**

In a multicenter sample of 278 adolescents with MDD, the prevalence of IA symptoms in adolescents with MDD was 46.8% (95% CI: 40.9–52.7%). Logistic regression analysis showed that patients with more severe depressive symptoms (odds ratio [OR] = 1.05, 95% CI: 1.03–1.08), those living in a rural area (OR = 1.94, 95% CI: 1.15–3.27), and those with poor academic performance (OR = 2.90, 95% CI: 1.42–5.95) were more likely to have IA symptoms. After controlling for confounding factors, patients with IA symptoms had significantly poorer QOL in the physical, psychological, and environmental domains than those without IA symptoms.

**Conclusion:**

IA symptoms are common in adolescents with MDD and appear to be associated with clinical symptoms. We could not infer a causal relationship between IA and depression because this was a cross-sectional study. Considering the positive association between IA symptoms and lower QOL, screening for IA symptoms should be conducted, and effective measures should be implemented for adolescents with MDD.

## Introduction

Internet addiction (IA) generally refers to the behaviors of uncontrolled and persistent use of the internet, which can lead to individuals’ distress and social avoidance (1). IA symptoms are prevalent among adolescents and have received extensive attention from researchers around the world (2–4). A systematic review and meta-analysis reported that the prevalence rate of IA symptoms ranged from 0.6 to 50% worldwide, with a pooled prevalence of 2.0% in children and adolescents (5). Previous studies showed that the prevalence of IA symptoms was 26.8–32.5% among adolescents in China (6, 7).

Previous studies suggested a possible link between depression and addictive behaviors. People with higher levels of depression are more likely to have problem gambling (8) and compulsive buying (9). Young adults with compulsive sexual behavior have more depressive symptoms than those without compulsive sexual behavior (10). Many studies have found that IA symptoms in children and adolescents are associated with psychiatric problems, including autism spectrum disorder (11), attention-deficit/hyperactivity disorder (ADHD) (12), and somatization/obsessive-compulsive symptoms (13). A previous study also found a bidirectional relationship between IA symptoms and depression in adolescents (14). Some neurobiological correlates [e.g., lower levels of oxytocin (15, 16), and the same biogenetic basis with “short” alleles of the 5HTTLPR polymorphism (17, 18)] can explain the clinical association between IA symptoms and depression.

Internet addiction symptoms negatively impact the academic performance and development of adolescents and may even lead to violence and crime (7, 19). These symptoms are also associated with lower quality of life (QOL) (20–22). A longitudinal survey found that more severe IA symptoms at an earlier time can predict higher levels of depression in adolescents and young adults later in life (23). Additionally, a recent study found that adolescents with IA symptoms were 2.2 times more likely to have suicidal ideation than those without IA symptoms (24). Targeting and managing IA symptoms among adolescents with major depressive disorder (MDD) at an early stage could potentially lead to improvements in psychological health and well-being, and a reduction of suicidality (25). There is a need to reveal the characteristics of IA symptoms in adolescents with MDD, which would facilitate the development of a new therapeutic target for the management of this patient population.

A study in Turkey found that the prevalence of IA symptoms in adolescents with MDD during the first assessment in the outpatient clinic was 30%, and the symptoms were associated with hopelessness (25). Another study in China reported that the prevalence in clinically stable adolescents with MDD was 36.9% (2). However, none of these studies explored the clinical correlates of IA symptoms in adolescents with MDD, such as the severity or duration of depression, or their association with QOL. Thus, the present study aimed to investigate the prevalence and clinical correlates of IA symptoms and their association with QOL in adolescents with MDD in China.

## Materials and Methods

### Study Design and Participants

This cross-sectional study was conducted in three general hospitals (Chaohu, Bengbu, and Bozhou) and four psychiatric hospitals (Hefei, Ma’anshan, Suzhou, and Fuyang) in Anhui Province, China, from January to July 2021. Participants were consecutively recruited from psychiatric outpatients and inpatients in these hospitals if they met the following criteria: (1) aged between 12 and 18 years; (2) had a diagnosis of MDD assessed by two psychiatrists using a structured clinical interview for DSM-V. Patients who met the DSM-V criteria for a diagnosis of other psychiatric or neurological disorders and/or intellectual disability were excluded.

All eligible participants and their guardians provided written consent forms after being informed of the method and purpose of the study. The Medical Ethics Committee of Chaohu Hospital of Anhui Medical University approved the study protocol (202009-kyxm-04) before the study.

### Data Collection and Measurements

Under the guidance of trained investigators, all adolescents participated in an interview that involved their guardians’ participation. A predesigned questionnaire was used to collect sociodemographic characteristics of the participants, including age (years), gender (male/female), living area (urban/rural) (26), geographical location (northern, central, or southern Anhui Province), academic performance (good, fair, or poor) (27), one-child status (a reply of “yes” or “no” to the question “Are you the only child in the family?”), and duration of illness (months). Specifically, the duration of illness was defined as the time range from the first onset of clinical symptoms to the time of participation in the evaluation (28). In this study, the Internet Addiction Test (IAT) was used to assess IA symptoms (29), which has been widely used in various populations worldwide, and the Chinese version has been validated among Chinese adolescents (Cronbach’s α coefficient = 0.93) (30). The scale included 20 questions about the actual frequency of online and related scenes, with an individual score ranging from 1 to 5 and a total score that ranged from 20 to 100. The adolescents with an IAT total score ≥50 were defined as “having IA” (7, 31–33), and the split-half reliability and Cronbach’s α coefficient were 0.86 and 0.90, respectively (31). In addition, the Center for Epidemiologic Studies of Depression Symptom Scale (CES-D) was used to assess the severity of depressive symptoms in patients with individual item scores of 0 to 3 (34). Furthermore, the Chinese version of the World Health Organization Quality of Life Brief version (WHOQOL-BREF) was used to evaluate QOL in the physical, psychological, social, and environmental domains (35, 36).

### Statistical Analysis

SPSS software version 23.0 (SPSS Inc., Chicago, Illinois, United States) was used for statistical analyses. Demographic and clinical data are presented as means, standard deviations (SDs), and frequency distributions (%). We compared sociodemographic and clinical variables between participants with and without IA symptoms using independent-samples *t*-tests for continuous variables with a normal distribution, Mann–Whitney U tests for continuous variables with a non-normal distribution, and chi-square tests for categorical variables. Analysis of covariance (ANCOVA) was used to compare QOL scores between groups with and without IA symptoms after controlling for the demographic and clinical variables that significantly differed between the two groups. Binary logistic regression analysis with the “Enter” method was used to examine the independent correlates of IA symptoms by treating the variables with significant group differences as independent variables and the presence of IA symptoms as the dependent variable. We calculated the sample size using PASS version 11.0 (NCSS Statistical Software, Kaysville, UT, United States) with the relevant parameters: the estimated prevalence of IA symptoms (30%), confidence level/1-α (0.95), and acceptable error (0.06). A minimum sample size of 239 was needed. Statistical significance was evaluated by a threshold of *P* < 0.05 (two-tailed).

## Results

### Participant Characteristics

The sociodemographic and clinical data of adolescents with MDD are presented in Table 1. Of 300 adolescents who were invited to participate in this study, 278 completed the evaluation and were included in the analysis, indicating a participation rate of 92.7%. The adolescent sample included 74 males (26.6%) and 204 females (73.4%). The mean age was 15.26 ± 1.70 years. Of these adolescents, 114 (40.3%) were the only child in their families, and 128 (46.0%) were living in a rural area.

**TABLE 1:** Demographic and clinical characteristics of participants with and without internet addiction symptoms

Variables	Whole sample (n=278)		Internet addiction (n=130)		No internet addiction (n=148)		Statistics	
	Mean	SD	Mean	SD	Mean	SD	Z/t	*P*
Age	15.28	1.71	15.28	1.58	15.29	1.81	-0.224*^a^*	0.822
BMI	21.18	4.08	21.12	4.30	21.24	3.89	-0.563*^a^*	0.573
CES-D total score	36.27	13.24	40.53	10.91	32.52	13.98	-4.791*^a^*	**<0.001**
Duration of illness	20.04	18.29	19.15	16.13	20.81	20.01	-0.290*^a^*	0.772
Physical QOL	11.16	2.32	10.33	1.93	11.90	2.39	5.945	**<0.001**
Psychological QOL	8.23	3.00	7.13	2.29	9.21	3.21	-5.908*^a^*	**<0.001**
Social QOL	11.32	3.75	10.72	3.91	11.85	3.54	-2.647*^a^*	**0.008**
Environmental QOL	11.19	2.64	10.36	2.41	11.91	2.62	5.125	**<0.001**

	***N***	***%***	***N***	***%***	***N***	***%***	***χ2***	***P***

Male	74	26.6	30	23.1	44	29.7	1.568	0.210
Living in rural area	128	46.0	69	53.1	59	39.9	4.863	**0.027**
Geographical location							1.517	0.468
Northern Anhui	71	25.5	37	28.5	34	23.0		
Central Anhui	173	62.2	76	58.5	97	65.5		
Southern Anhui	34	12.2	17	13.1	17	11.5		
One-child family	114	41.0	48	36.9	66	44.6	1.684	0.194
Academic performance *^b^*							12.961	**0.002**
Good	120	43.2	44	33.8	76	51.4		
Fair	105	37.8	51	39.2	54	36.5		
Poor	53	19.1	35	26.9	18	12.2		
Antidepressants	173	62.2	78	60.0	95	64.2	0.517	0.472
Antipsychotics	45	16.2	22	16.9	23	15.5	0.098	0.755

*^a^Mann–Whitney U test; ^b^An original 5-point Likert scale was used: good (verygood or good), fair, and poor (very poor or poor). Bold values: P< 0.05; BMI, body mass index; CES-D, Center for Epidemiologic Studies of Depression Symptom Scale; QOL, quality of life.*

### Comparisons Between Groups With and Without Internet Addiction Symptoms

The prevalence of IA symptoms was 46.8% (95% CI: 40.9–52.7%) in the total sample. There was no significant difference in the prevalence between males and females (40.5% vs. 49.0%, respectively; *P* = 0.210). The adolescents with IA symptoms had more severe depressive symptoms, worse academic performance, and a higher proportion of living in a rural area than those without IA symptoms (all *P* < 0.05). In addition, the adolescents with IA symptoms had lower scores in all domains of QOL than those without IA symptoms. ANCOVA showed that differences in physical (*F* = 8.49, *P* = 0.004), psychological (*F* = 10.30, *P* = 0.001), and environmental QOL scores (*F* = 5.03, *P* = 0.026) between the two groups remained significant after controlling for CES-D total score, living area, and academic performance. However, the group difference in the social domain was not statistically reliable (*F* = 0.02, *P* = 0.886).

### Independent Correlates of Internet Addiction Symptoms

The results of multiple logistic regression analysis with the independent correlates associated with IA symptoms are presented in Table 2. We found that more severe depressive symptoms (OR = 1.05, 95% CI: 1.03–1.08, *P* < 0.001), living in a rural area (relative to an urban area) (OR = 1.94, 95% CI: 1.15–3.27, *P* = 0.013), and poor academic performance (compared to good academic performance) (OR = 2.90, 95% CI: 1.42–5.95, *P* = 0.004) were independently associated with IA symptoms in the adolescents with MDD.

**TABLE 2:** Demographic and clinical variables independently associated with internet addiction symptoms by binary logistic regression analysis.

Variables	*P*	*OR*	95% *CI*	
			Lower	Upper
Living in rural area	**0.013**	1.94	1.15	3.27
CES-D total score	**<0.001**	1.05	1.03	1.08
Academic performance				
Good	—	Reference	—	—
Fair	0.075	1.67	0.95	2.95
Poor	**0.004**	2.90	1.42	5.95

*Adjusted R^2^ = 0.193. Bolded values: P < 0.05; CES-D, Center for Epidemiologic Studies of Depression Symptom Scale; OR: odds ratio; CI, confidence interval.*

## Discussion

In this study, we examined the prevalence and clinical correlates of IA symptoms and explored their associations with QOL in Chinese adolescents with MDD. We found that the prevalence of IA symptoms in the patient sample was 46.8% (95% CI: 40.9–52.7%), which is much higher than that in Chinese adolescents in extant studies. For example, a survey of 10,158 adolescents from Anhui Province showed that the prevalence of IA symptoms was 10.4% (37). A more recent study involving 31,954 adolescents in Beijing, a more developed area, reported a prevalence of 6.2% (38). Several notable factors could contribute to the high prevalence of IA symptoms in adolescents with MDD. First, adolescents with MDD often stay home from school, which provides much leisure time and less family supervision and enables young patients to easily access and indulge in using the internet (39, 40). Second, adolescents with MDD are more likely to indulge in the virtual cyber world instead of the real world, because they can have more fun, which counteracts the negative effects of depression (41).

Specifically, the prevalence rate of IA symptoms in our study was approximately 1.5 times the rates reported in adolescents with MDD during an initial assessment in a Turkish outpatient clinic (30%) (25) and in clinically stable adolescents with MDD in China (36.9%) (2). The discrepancy could be attributed to patient characteristics. This current study recruited adolescents with MDD, including not only stable patients but also those with more severe depressive symptoms. In light of our findings, adolescents with more severe depressive symptoms are more likely to have IA symptoms, which is consistent with previous findings in the school sample of Chinese adolescents (7, 42). In addition, adolescents with more severe depressive symptoms are prone to report having deficient social skills and seek social connections online (43). Excessive reduction in real-world social activities may further lead to IA symptoms (44). Importantly, for individuals who are addicted to the internet, isolation from the real world increases the severity of depressive and anxiety symptoms (45, 46). Costa et al. found that a lack of adequate sensory contact and physical feedback in online communication can bring about feelings of loneliness in adolescents and young adults (47), which may further contribute to depressive symptoms. Given that depressive symptoms and IA symptoms in adolescents reinforce each other, improving depressive symptoms should be considered an effective intervention as a priority for reducing the risk of IA symptoms. In the meantime, the adoption of interventions for IA symptoms could be beneficial for depressive symptom mitigation.

In addition, we found that IA symptoms were associated with poor academic performance in adolescents with MDD, which replicated previous findings in general adolescents (7, 37) and college students (48, 49). Existing evidence shows that poor academic performance and IA symptoms interact with each other in a vicious cycle. Adolescents with poor academic performance feel pessimistic about their future and give up their studies, as they cannot meet the expectations of their parents and teachers. This could lead to their excessive use of the internet and development of IA symptoms. In addition, adolescents with IA symptoms often spend limited time on course work, thus resulting in a consequent decline in academic performance. A survey of middle-school students in northern Taiwan revealed that the patterns of internet use affected students’ academic performance, as online socializing and gaming led to poorer exam performance a year later (50).

Moreover, we found that adolescents living in rural areas were more likely to report IA symptoms than those living in urban areas. This difference might be closely linked to their differing living conditions and habits. For adolescents living in rural areas, the dominant way to spend time is through online socializing, gaming, and shopping due to the lack of diverse recreational activities. Previous studies have shown that without parental supervision, adolescents left alone in rural areas have unrestricted access to the internet, which could further enhance IA symptoms (40, 51). In contrast, a previous study reported a lower rate of IA symptoms among adolescents in rural areas than urban areas, which was attributed to the fact that adolescents in rural areas had less access to the internet (52). However, with increasing availability of the internet in China in recent years, access to and the convenience of the internet in rural areas have become comparable to those in urban areas (53). Therefore, to reduce the high rate of IA symptoms and limit their adverse effects in rural adolescents, there is a strong need to take effective measures to reduce excessive internet use, such as installing more recreational facilities (e.g., basketball courts, table tennis equipment) in rural areas, establishing regular physical activity schedules, and strengthening parental supervision of internet use among adolescents.

In this study, adolescents with IA symptoms had lower levels of QOL in all domains than those without IA symptoms, which is consistent with the findings in a large-sample study with 12,285 adolescents in Spain (54) and a multicenter study in China (22). First, excessive internet use can lead to multiple physical health issues, such as headache (55), oral diseases (56), musculoskeletal pain (57), obesity (58, 59), hypertension (60), and eating disorders (61). Moreover, adolescents with IA symptoms often experience distress symptoms, familial conflicts, and social withdrawal (62). Interestingly, after controlling for the severity of depressive symptoms, living area, and academic performance, the difference in social QOL between the two groups disappeared. This finding is consistent with a previous study of adolescents in China (22). Our interpretation is that IA symptoms are associated with social QOL but may not be independent from sociodemographic and clinical factors in adolescents with MDD. Collectively, these findings imply that improving IA symptoms could increase QOL among adolescents with MDD.

Several limitations in this study should be noted. First, because the current design was cross-sectional, the direction of causality between IA symptoms and clinical factors or QOL in adolescents with MDD might not be adequately established. Second, although the prevalence of IA symptoms among adolescents has been widely reported, there was no age- and gender-matched healthy control group in this study. As such, we were unable to make direct comparisons of IA symptom prevalence and QOL between MDD adolescents and those in school or community samples. Third, depressive symptoms were assessed by the CES-D, a self-report tool, rather than an objective measure for depressive symptoms, such as the Hamilton Depression Rating Scale (HAMD). Finally, relevant factors associated with IA symptoms, such as parental internet use, economic conditions, and social support, were not examined. These limitations could be addressed by future studies.

## Conclusion

In summary, IA symptoms are common in adolescents with MDD, particularly in those with more severe depressive symptoms, living in a rural area, and with poor academic performance. Given the negative impact of IA symptoms on QOL in adolescents with MDD, effective and proactive interventions, including regular screening, improving depressive symptoms, enriching recreational activities, and strengthening parental supervision, should be undertaken in this population.

## Data Availability Statement

The data used for this study are available from the corresponding author on reasonable request.

## Ethics Statement

The studies involving human participants were reviewed and approved by the Medical Ethics Committee of Chaohu Hospital of Anhui Medical University. Written informed consent to participate in this study was provided by the participants’ legal guardian/next of kin.

## Author Contributions

HL: study design. SW, LX, JW, XY, YS, XW, XL, and YH: collection, analyses, and interpretation of data. SW, LX, and JW: drafting of the manuscript. FG and HL: critical revision of the manuscript. All authors approval of the final version for publication.

## Conflict of Interest

The authors declare that the research was conducted in the absence of any commercial or financial relationships that could be construed as a potential conflict of interest.

## Publisher’s Note

All claims expressed in this article are solely those of the authors and do not necessarily represent those of their affiliated organizations, or those of the publisher, the editors and the reviewers. Any product that may be evaluated in this article, or claim that may be made by its manufacturer, is not guaranteed or endorsed by the publisher.
